# Determination of acceptable Hounsfield units uncertainties via a sensitivity analysis for an accurate dose calculation in the context of prostate MRI-only radiotherapy

**DOI:** 10.1007/s13246-023-01333-5

**Published:** 2023-10-10

**Authors:** Hilda Chourak, Anaïs Barateau, Peter Greer, Caroline Lafond, Jean-Claude Nunes, Renaud de Crevoisier, Jason Dowling, Oscar Acosta

**Affiliations:** 1 Univ Rennes, CLCC Eugène Marquis, INSERM, LTSI - UMR 1099, Rennes, France; 2 CSIRO Australian e-Health Research Centre, Herston, QLD Australia; 3 School of Mathematical and Physical Sciences, University of Newcastle, Newcastle, NSW Australia; 4 Radiation Oncology, Calvary Mater Newcastle Hospital, Newcastle, NSW Australia

**Keywords:** Sensitivity analysis, Quality assurance, Synthetic-CT, MRI-only radiotherapy, Prostate cancer

## Abstract

Radiation therapy is moving from CT based to MRI guided planning, particularly for soft tissue anatomy. An important requirement of this new workflow is the generation of synthetic-CT (sCT) from MRI to enable treatment dose calculations. Automatic methods to determine the acceptable range of CT Hounsfield Unit (HU) uncertainties to avoid dose distribution errors is thus a key step toward safe MRI-only radiotherapy. This work has analysed the effects of controlled errors introduced in CT scans on the delivered radiation dose for prostate cancer patients. Spearman correlation coefficient has been computed, and a global sensitivity analysis performed following the Morris screening method. This allows the classification of different error factors according to their impact on the dose at the isocentre. sCT HU estimation errors in the bladder appeared to be the least influential factor, and sCT quality assessment should not only focus on organs surrounding the radiation target, as errors in other soft tissue may significantly impact the dose in the target volume. This methodology links dose and intensity-based metrics, and is the first step to define a threshold of acceptability of HU uncertainties for accurate dose planning.

## Introduction

External beam radiation therapy (EBRT) involves the application of high-energy x-ray beams from multiple directions, depositing energy (dose) within a tumour to destroy cancer cells. EBRT is a well-established treatment modality for localised prostate cancer. Until recently, treatment has traditionally been planned based on Computed Tomography (CT), with Magnetic Resonance Imaging (MRI) also acquired for diagnostic information. For prostate cancer, MRI has added significant value to EBRT due to its superior soft tissue contrast which results in the improved accuracy of manual labelling of the target volume (the prostate gland) and nearby organs at risk (bladder, rectum, bones). This improved accuracy may reduce the risk of toxicity in healthy tissue [1, 2].

The deployment of MRI-only radiotherapy (RT) provides greater efficiency and accuracy in the clinical workflow by bypassing the MR to planning CT registration step and removes the need for an extra CT scan. This justifies the increasing worldwide deployment of dedicated MRI scanners and MRI-linear accelerator (MRI-linac) hybrid machines for treatment delivery, the latter also allows for better patient positioning and tumour targeting [3]. However, MRI does not provide information on the electron density of tissues, which is necessary for dose calculation. Synthetic-Computed Tomography (sCT) generation is thus a critical component of MRI-only RT workflows.

Currently, sCT images are assessed against a ground truth CT in two ways: image and dose [4]. The first method involves a comparison of Hounsfield Units (HU) [5, 6]. The most commonly used metrics are full reference intensity-based and include mean absolute error (MAE), mean error (ME) and peak signal-to-noise ratio (PSNR). Perception-based models like the structural similarity (SSIM) may also be assessed [7, 8], and more specifically the multiscale SSIM (MS-SSIM) [9]. These metrics result in global or organ-wise values, but local errors such as air incorrectly included within an organ may have an impact on treatment delivery and may not be identified with a global metric. For sCT in the pelvic area, the HU uncertainties are typically observed in the cortical bone and rectum when air pockets are present [10, 11].

The quality of sCT images are also assessed by the dose accuracy. For the different EBRT treatment techniques such as intensity-modulated radiation therapy (IMRT) or volumetric modulated arc therapy (VMAT), the beams cross several healthy tissues before reaching the target. Errors in these beams’ trajectories will have consequences on the dose delivered to the target. Most of the sCT generation literature describe dosimetric endpoints such as gamma analysis and dose-volume histograms (DVH) metrics [12]. These measures give an insight of the overall dose distribution accuracy on the sCT. A previous study proposed a voxel-wise statistical analysis strategy to locally assess sCT generation approaches in image and dose domains [13], but no correlation was made between both. Choi et al. [14] investigated the correlation between image metrics as a global value (computed within the body contour) and dose accuracy in the target volume and proposed a water equivalent depth method as a metric. However, no information was given on the origin of dosimetric errors. Generated images must be sufficiently correct to ensure accurate dose planning in the tumour area. So, determining the origin of local erroneous dose will allow focusing on the most meaningful HU error and provide thresholds of HU uncertainties acceptability.

The aim of this study is to investigate the correlation between localised HU errors and dose at the centre of the target volume, here the prostate. To do so, a sensitivity analysis (SA) was performed, by applying the Morris screening method [15]. An SA is designed to quantify the effect of parameters on the output [16]; in this study, the effect of HU error on the dose distribution at the isocentre (centre of the prostate).

Several SA methods exist and can be classified in two types: local and global. Local methods allow for the examination of the model at a specific point in the input space. Most of these approaches induce a low computational cost. However, they do not give an indication of interactions between parameters or on the linearity of their effects. Global methods measure the sensitivity in several points in the input space and highlight the type of effect and the possibility of interactions [17]. SA has previously been applied to assess the ability of quality assurance protocols to detect events affecting MRI in RT [18], or to evaluate the sensitivity of electron dose calculation with respect to stopping power and transport coefficients [19].

In this study, a global one-at-a-time (OAT) approach, the Morris screening method, has been chosen to identify the impact of uncertainties in synthetic-CT on the isodose. The Morris method has previously demonstrated its ability to simplify models predicting biochemical recurrence after radiotherapy [20] by discarding parameters with a low impact on the output. Applying this methodology to sCT for MRI-only RT is the first step in the definition of thresholds of acceptability of HU errors in sCT for safe MRI-only RT practice.

## Materials and methods

Two experiments have been conducted to determine the errors in sCT that are more likely to affect the dose at the isocentre. First, the errors have been assessed in terms of HU number, volume, and location by adding an artefact in the reference CTs. Spearman correlation coefficient (SCC) between error features (intensity, volume, location) and dose at the isocentre were computed. While the SCC indicates if the different features have a monotonic impact on the dose, the SA will help to classify the features according to their influence on the output and give information on the linearity and or interaction between factors.

In a second phase, we focused on the impact of errors in specific anatomical location by changing the mean intensity in the bladder, rectum, bones, prostate and in the remaining soft tissues.

### Dataset

Data of 39 patients with localised prostate cancer aged 58 to 78 years were used in this study. Ethics approval for the study protocol was obtained from the local area health ethics committee, and informed consent was obtained from all patients. For each patient, a CT scan was acquired on a GE LightSpeed RT or a Toshiba Aquilion, (256 × 256 × 128 matrix with a voxel size of 1.17 mm × 1.17 mm × 2.5 mm or 2.0 mm). Bones, bladder, rectum, and prostate were manually delineated by experts.

### Sensitivity analysis: Morris screening method

**Fig. 1:**
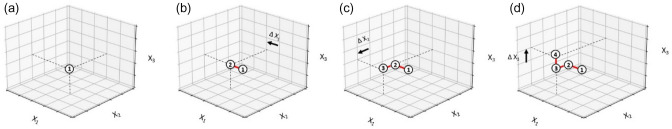
Example of a trajectory, for the evaluation of the influence of K = 3 factors. First, one point is randomly selected in the 3-dimensional space (**a**). Then, three other points are created by changing one parameter value at a time (**b**, **c** and **d**)

The Morris screening method is a randomised OAT global SA. The parameters are modified individually, and cover a K-dimensional cube, with K representing the number of factors (Fig. 1).

Feature values were generated using the Sensitivity R package [21] and were randomly assigned to efficiently cover the K-dimensional space. Elementary effects (EE) given by (1) are calculated to assess the effect of the ${X}_{i}$ factor variation on the output. The model is evaluated $N=R\times \left(K+1\right)$ times for each $j$ patient, with $R$ the number of repetitions, i.e. the number of EE computed per factor.

$$\begin{array}{*{20}c} {\begin{array}{*{20}c} {EE_{{i,j}} = \frac{{f_{j} \left( {X_{1} , \ldots ,X_{i} + \Delta _{i} , \ldots ,X_{K} } \right) - f_{j} \left( {X_{1} , \ldots ,X_{i} , \ldots ,X_{K} } \right)}}{{\Delta _{i} }}} \\ \end{array} .} \\ \end{array}$$

${\varDelta }_{i}$ is the discrete variation of the parameter.

For each factor and each patient, the mean${\mu }_{i,j}$ (2) of EE, the standard deviation ${\sigma }_{i,j}$ (3), and the mean of the absolute values of the EE ${\mu *}_{i,j}$ (4) are computed to summarise the EE and thus estimate the global sensitivity in the output space [22]. ${\mu *}_{i,j}$ is used to solve the effect of opposite signs for non-monotonic functions.

$$\begin{array}{*{20}c} {\begin{array}{*{20}c} {\mu _{{i,j}} = \frac{{\sum _{{r = 1}}^{R} EE_{{i,j}}^{r} }}{R}} \\ \end{array} } \\ \end{array} ,$$

$$\begin{array}{*{20}c} {\begin{array}{*{20}c} {\sigma _{{i,j}} = \frac{{\sum _{{r = 1}}^{R} \left( {EE_{{i,j}}^{r} - \mu _{{i,j}} } \right)}}{R}} \\ \end{array} } \\ \end{array} ,$$

$$\begin{array}{*{20}c} {\begin{array}{*{20}c} {\mu *_{{i,j}} = \frac{{\sum _{{r = 1}}^{R} \left| {EE_{{i,j}}^{r} } \right|}}{R}} \\ \end{array} } \\ \end{array} .$$

To illustrate the impact of the parameters on the output, the Euclidean distance of each point to the origin $$\left(\mu *0,\sigma =0\right)$$$${D}_{i}=\sqrt{{{\mu *}_{i,j}}^{2}+{{\sigma }_{i,j}}^{2}}$$ has been calculated [23].

**Fig. 2:**
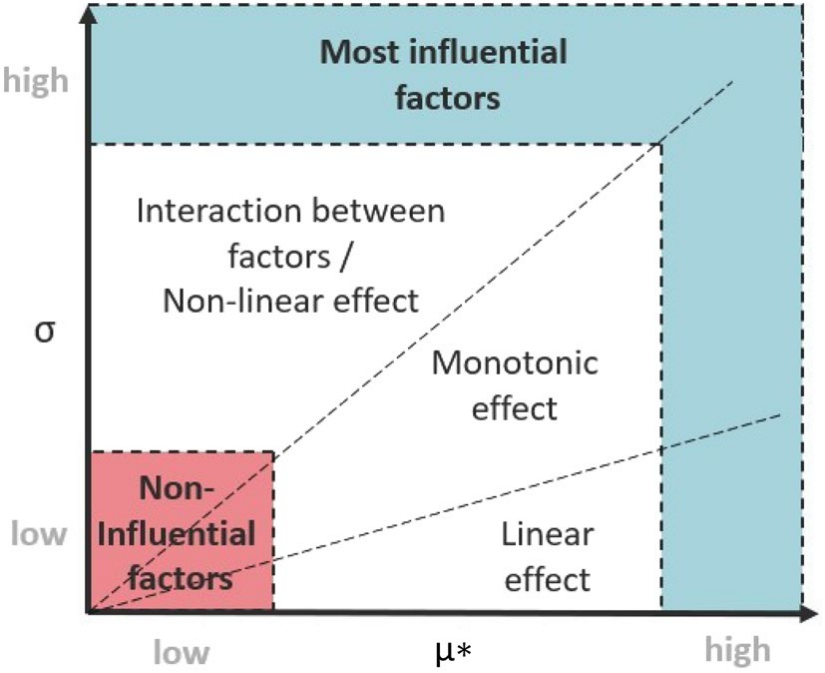
Classification of parameters according to the mean of the absolute elementary effects (µ*) and their dispersion (σ)

Low $\mu *$ and $\sigma$ indicate an insignificant impact for a chosen factor, and high $\mu *$ and/or $\sigma$ stand for significant impact. High value of $\sigma$ compare to $\mu *$ indicates a factor involved in interaction with others factors or whose effect is non-linear (Fig. 2).

In this study, the Morris screening approach aimed to emphasise the impact of localised HU errors on dose calculation, according to:

- descriptive characteristics of the error (intensity, size and location),
- mean intensity within the organs.

These two approaches are described in the experiment’s sections below.

#### Experiment 1

The first experiment aimed to assess the impact of error according to 3 factors: intensity, size and location. To achieve this, an artefact with various combinations of these three parameters has been added to the 39 planning CTs. The artefact was built as follows:

- HU variation, from − 250 to + 250 HU.
- Distance to the isocentre, from 0 to 100 mm. The artefact displacement followed one of the beams’ axis.
- Diameter of the artefact, from 2 to 50 mm.

The model has been evaluated 200 times for each patient: $N=R\times \left(K+1\right),$ with $R=50$ repetitions, and $K=3$ factors (intensity, distance, size), resulting in 7800 simulations.

The Spearman correlation coefficient (SCC) has also been computed in this experiment. This is a nonparametric measure of statistical dependence of ranking between two variables.

An SCC close to − 1 or 1 denotes a strong correlation, while an SCC close to 0 illustrates a weak relationship.

To compute the SCC for each error features, the following parameters have been defined:

- For the effect of changes in HU, the step was set to 25 HU. The diameter of the artefact was fixed to 50 mm and its centre aligned to the isocentre, allowing for complete coverage of the target and ensuring a homogeneous distribution of the dose within the error volume.
- For the effect of distance, the step was set to 10 mm, with an error fixed at + 200 HU and a size of 50 mm. The displacement followed a beam axis, minimizing the impact of the dose on the result. (For the error to have consequences on the dose at the isocentre, it must be encountered by one of the beams delivering the treatment).
- For the effect of size, the error was fixed at + 200 HU and located at 30 mm from the isocentre. This location corresponds approximately to the rectum, where high HU variation can be observed due to the difficulty of predicting air pockets.

2145 images were generated to compute the SCC.

#### Experiment 2

Errors in sCT are more likely to be evaluated in terms of mean HU error within the body or per organs [24–27]. So, in this experiment, mean intensity changes in the following locations have been applied in order to assess their potential impact on the dose:

- Bladder (from − 100 to + 100 HU),
- Rectum (from − 1000 to + 200 HU),
- Bones (from − 500 to + 500 HU),
- Prostate (from − 100 to + 100 HU),
- Remaining soft tissue (from − 100 to + 100 HU).

Remaining soft tissue volumes are generated by subtraction of bone, bladder, prostate and rectum volumes from the body contour. The model was evaluated 240 times for each patient $R=40$ repetitions, and $K=5$ factors), resulting in 9360 simulations. Higher threshold has been defined for bone and rectum, according to the difficulty for a sCT generation method to predict HU in these locations. Especially for the rectum, where the presence of gas (− 1000 HU) is uncertain.

### Dose planning

IMRT with 7 beams (photons of 6 MV) was planned for 39 fractions (2 Gy per fraction) on reference CT images using a dose grid resolution of 3 × 3 × 3 mm with MatRad [28], an open-source software for radiation treatment planning developed for research purposes [29–31]. The beam parameters used to compute the dose on the CT were then copied to calculate the dose on each modified CTs.

**Fig. 3:**
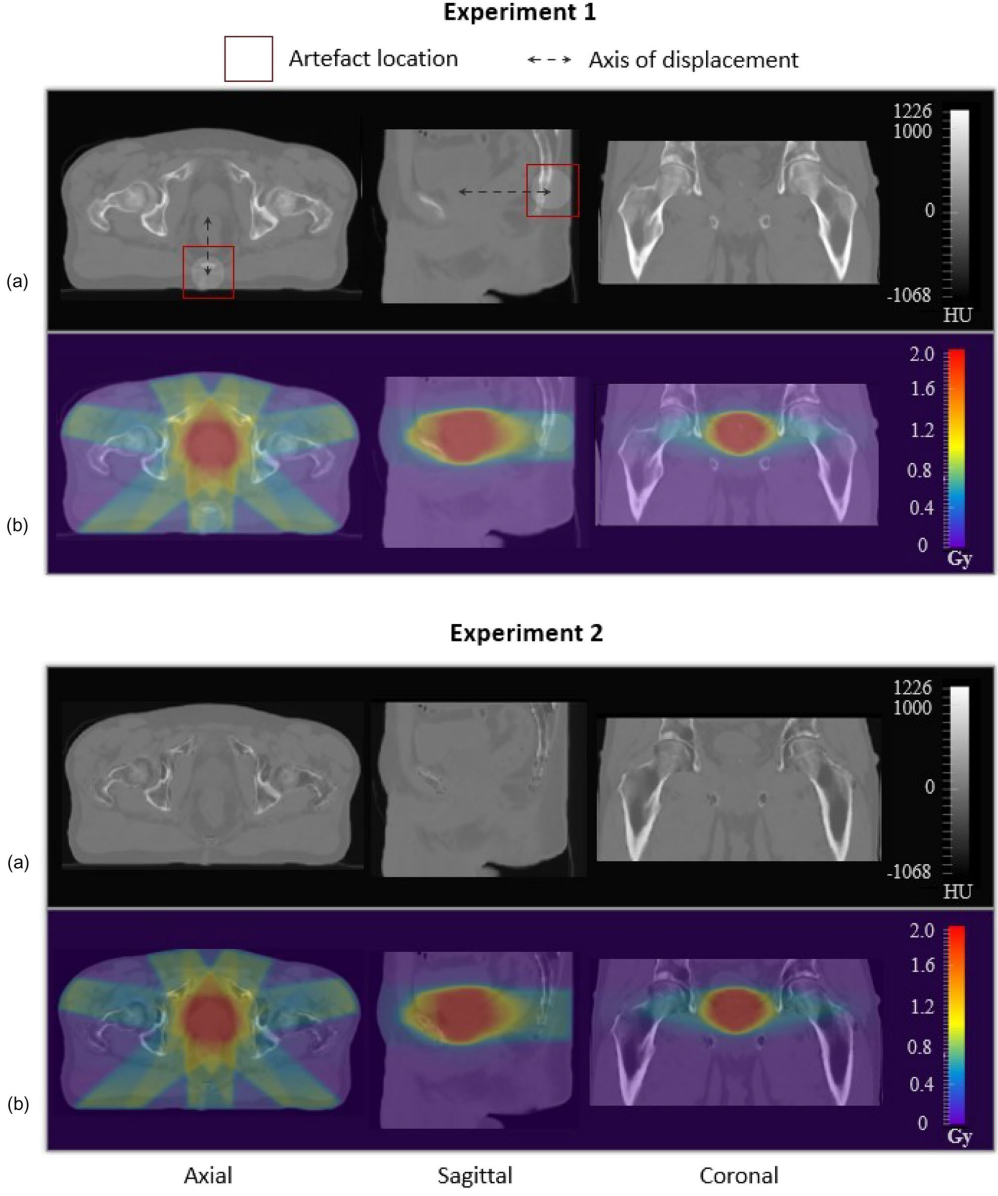
Example of a Modified CT (**a**) and its corresponding dose distribution overlaid (**b**) for both experiments. For Experiment 1, the error volume is visible in the red square, and the dotted arrow represent its axis of displacement. A modified image of the same patient has been randomly selected to illustrate Experiment 2. Here, 100 HU were added in the bladder, 66.6 HU in the rectum, 55.5 HU in the prostate, and 78 HU in the remaining soft tissue. 146.5 HU were subtracted in the bones

Figure 3 presents examples of modified CT and their corresponding dose used in this study.

## Results

### Experiment 1

**Fig. 4:**
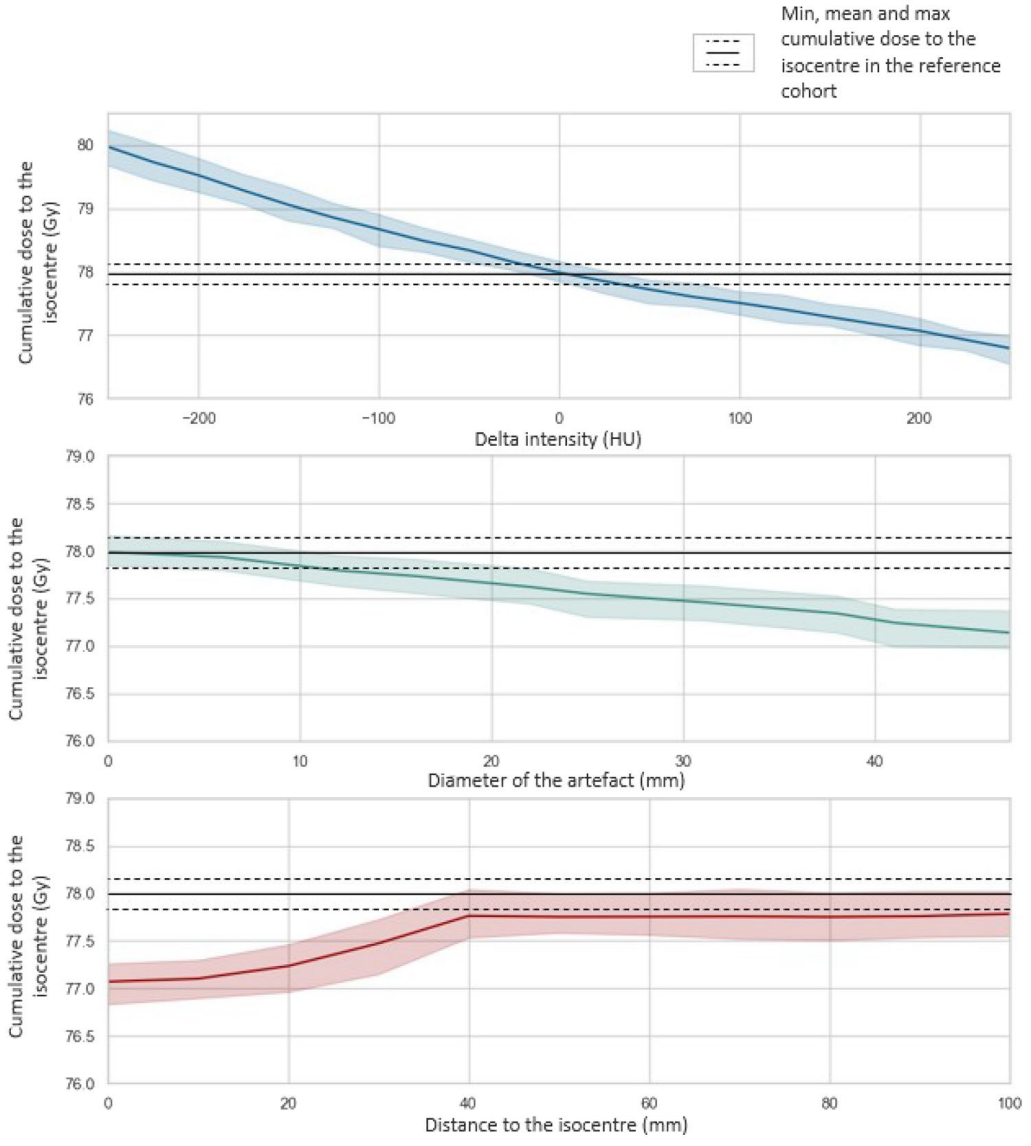
Experiment 1: impact of the error (in terms of intensity in blue, size in green and distance in red) on the dose distribution at the isocentre

The relationship between the 3 error features and the isodose appear to be monotonic, with an SCC of − 0.99 for the intensity variation, − 0.95 for the size and 0.73 for the distance. As shown in Fig. 4, an overestimation of HU will reduce the dose distributed in the target, while an under estimation will result in a higher dose delivered at the isocentre. Also, there is an important interaction between the size of the volume error and the beams delivering the dose. As the amount of this volume within the beam increases there are greater impacts on the treatment. An artefact with a diameter of 30 mm will decrease the dose in the target of 0.5 Gy in average. As the error is fixed at + 200HU to assess the impact of the size, the dose distribution will decrease in this graph.

Regarding the distance, the closer is the volume from the isocentre, the more important is the impact of the error in this location. For all of the patient cohort, when the distance to the isocentre reaches 40 mm, the impact of the artefact starts to be constant, without reaching the prescribed dose (78 Gy). This might be explained by the variation of the dose going through the volume of error.

**Fig. 5:**
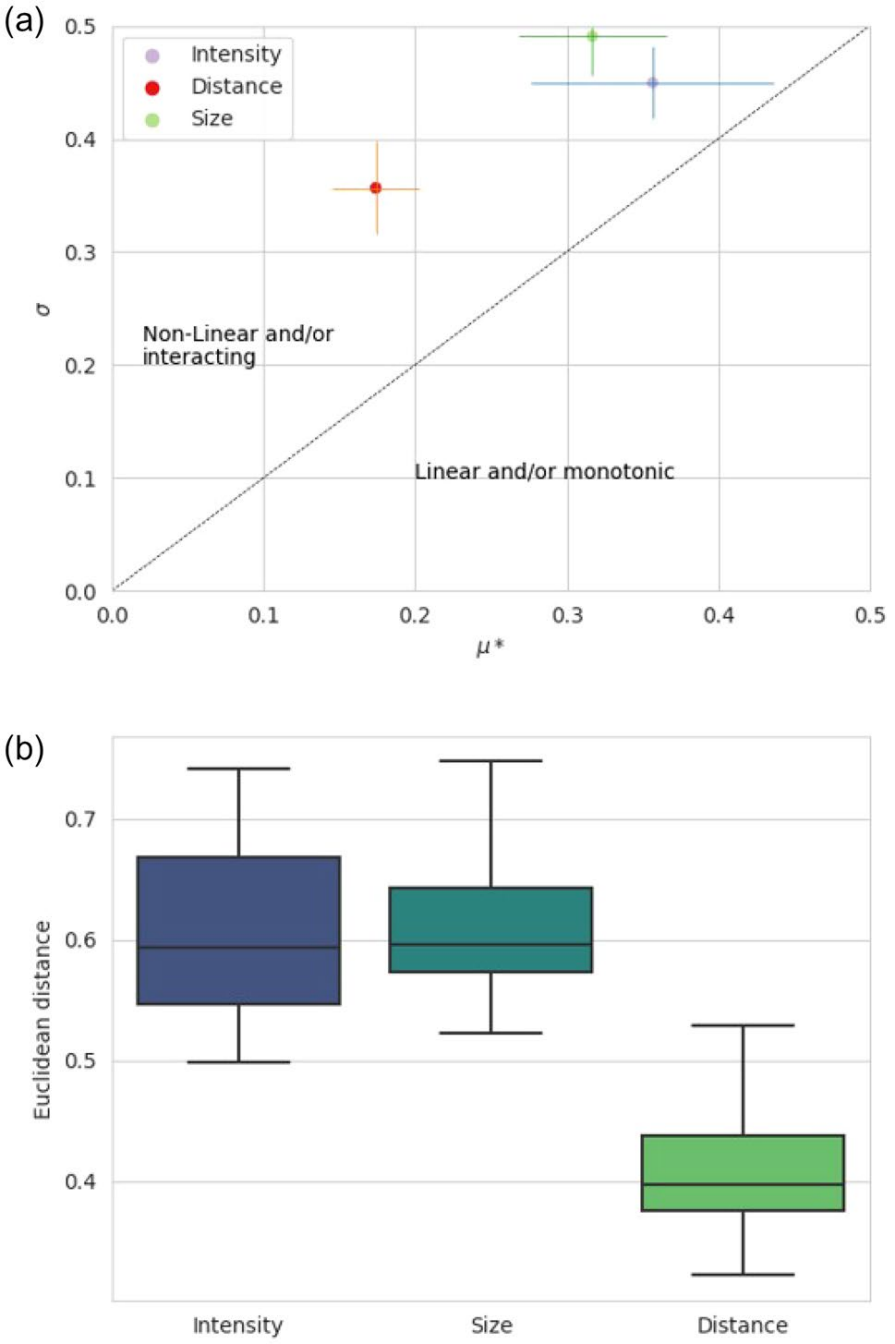
Morris screening results for the Experiment 1. **a** Mean of (µ*_i,j_, σ_i,j_) for each factor. The bars correspond to the standard deviation of µ*_i,j_ and σ_i,j_ across the patient cohort. **b** Euclidean distance of each point (µ*_i,j_, σ_i,j_) to the origin of the graph σ = f(µ∗) in descending order of importance

The SCC gives an insight of the effect of each parameter on the dose distribution, but this covers only a few possible combinations of factors compared to the Morris screening method. Figure 4a presents the results of the SA. $\sigma$ is superior to $\mu *$ for all the factors assessed: their effect on the dose distribution at the isocentre is thus non-linear/non-monotonic and/or they interact with each other. This figure also shows that intensity and size are the two most impactful parameters. This statement is confirmed by Fig. 4b, as the Euclidean distance to the origin of the graph is an indication of the influence of a factor on the output. Indeed, it shows that on average, the intensity and the size have both a similar impact on the output (Fig. 5).

### Experiment 2

**Fig. 6:**
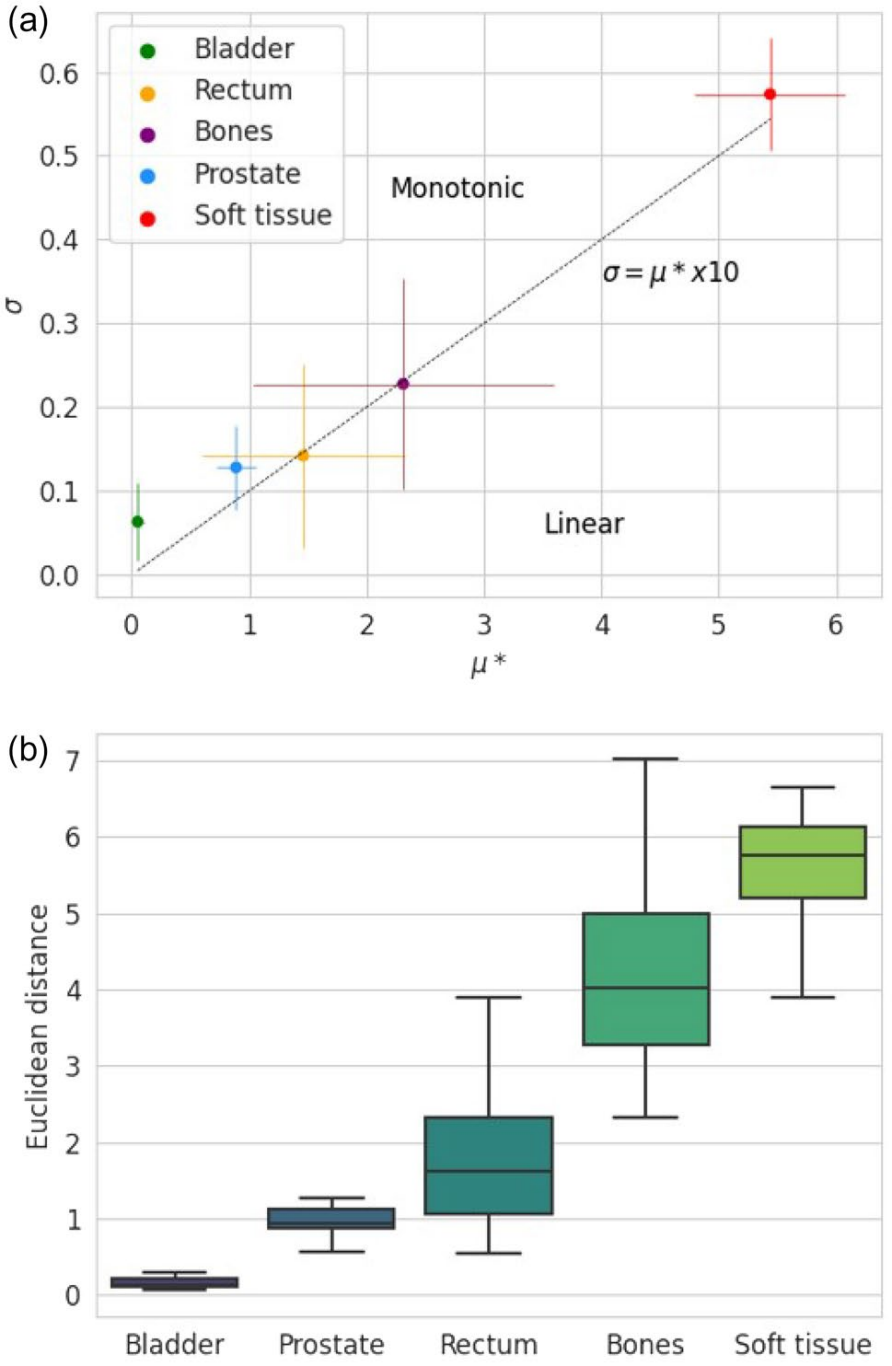
Morris screening results for Experiment 2. **a** Mean of (µ*_i,j_, σ_i,j_) for each factor. The bars correspond to the standard deviation of µ*_i,j_ and σ_i,j_ across the patient cohort. **b** Euclidean distance of each point (µ*_i,j_, σ_i,j_) to the origin of the graph σ = f(µ∗) in descending order of importance

Figure 6 shows that change in the bladder and prostate intensities do not implies significant change in the dose at the isocentre. The dose appeared to be more sensitive to errors in the bones and rectum. The standard deviation of and are more important for these anatomical locations, so change of intensity had a less constant impact across the patient cohort than for the bladder or prostate. Errors in the remaining soft tissue are the most impactful.

## Discussion

As different sCT generation methods will produce different and inhomogeneous HU uncertainties across the patient’s body [11, 13, 26], two experiments have been performed. The first one highlighted the sensitivity of the dose to changes in intensity and size of the volume of error. According to Fig. 4, the three features assessed had a monotonic effect on the output, and the Morris screening analysis demonstrated that the three parameters interact with each other.

The second experiment presented the result of the SA on organ-wise error. The variation of HU has been applied homogeneously across each structure to be consistent with the way the methods are usually assessed in the literature (mean error within the organs and the body contours).

Sensitivity to errors in the bones and rectum is less consistent across the patient cohort (Fig. 4) compared to errors in the bladder and prostate. This might be due to higher variability of size and HU in these structures, with the presence or absence of rectal gas, and different densities in the bone structure (cortical and spongy bones, with a variability in density across the population due to age and body mass [32]). The size of the bones, varying with size of the individual, would also depend on patient weight, where for a thin person an error in bone would have more influence as there is less soft tissue. In this experiment, unlike in the first one, the impact of the different parameters assessed tended to be linear. This might be explained by the consistency of the size and distance of each structure assessed, so the only changing factor is the variation of HU.

Some studies evaluated the dosimetric impact of HU to density curve variation. For example, in a previous study, Thomas et al. [33] reported a dosimetric error of 1.0% for a difference of 8.0% in bone electron density. Notable HU variations affect the accuracy of dose calculation [34, 35]. In case of HU to density curve error, the whole CT image is impacted for a given tissue. In this study, we focused on specific local area.

An absolute threshold of acceptability cannot be universally defined since it depends on each specific sCT generation method and treatment scenario. Therefore, it is recommended to apply this methodology to each clinical centre’s specific data. The obtained results are specific to the dose calculation algorithm, the number of beams crossed by the volumes and the amount of dose delivered in each of them. In this study, we assessed the effect of errors on IMRT dose plans, but other treatment techniques may be used in the clinic like VMAT, and stereotactic body radiation therapy (SBRT) [36], may result in different dose distributions across the body and thus will have a strong impact on the results. For particle therapy, the dose in the normal tissue outside the target volume is reduced [37], and the dosimetric impact due to misprediction in HU are likely to be larger. Different results are thus expected for proton and carbon ion therapy. Future work will investigate the other treatment techniques, with a more significant link with the sCT generation.

We focused on the dose at the isocentre, but changes in HU also have consequences on dose distributions in the organs at risk (bladder, rectum, femoral heads), leading to toxicity and inconvenient secondary effects such as chronic bladder inflammation. Therefore, future work will also explore the local influence of HU modification, using dose-volume histogram differences in each specific location.

In the pelvic area, the anatomy of the patient is subject to change due to variation of the bladder and rectal filling for example, which may have consequences on the accuracy of the treatment delivery [38, 39]. The method proposed in this paper could also be used to determine an acceptance criteria of organs motion during the treatment.

The methodology presented in this study can be adapted to each specific generation method, once the location of HU uncertainties has been identified, and the treatment plans defined. Deep-learning based sCT generation methods tend to be the most common [40], and more effective models should to be developed in the future. Aleatoric (data dependant) and epistemic (model dependant) uncertainties are specific to machine-learning models and can be assessed [41–43]. Including the impact of these uncertainties on the dose distribution during the learning process might be a way to create more clinically valid image generation.

## Conclusion

A sensitivity analysis was performed, allowing for determining the less influential HU errors on the dose distribution at the isocentre. sCT assessment should not only focus on delineated contours, and sparse error in the body contours should not be neglected. This study confirms the necessity to locally assess each generated sCT prior to its used in a clinical workflow, particularly in steep dose gradient area.

The main contribution of this paper is to provide a bridge between intensity-based metrics and dose, which are often used independently to assess the quality of sCT for EBRT. This approach can be used to generate clinical thresholds, and potentially model constraints, for both training and validation of sCT generation methods. The study is the first step in the definition of threshold of uncertainty acceptability in sCT to ensure accurate MRI-only RT.

## References

[1] Bruynzeel AME (2019). A prospective single-arm phase 2 study of stereotactic magnetic resonance guided adaptive radiation therapy for prostate cancer: early toxicity results. Int J Radiat Oncol Biol Phys.

[2] Kishan AU (2022). Magnetic resonance imaging-guided versus computed tomography-guided stereotactic body radiotherapy for prostate cancer (MIRAGE): Interim analysis of a phase III randomized trial. J Clin Oncol.

[3] Ng J (2023). MRI-LINAC: a transformative technology in radiation oncology. Front Oncol.

[4] Johnstone E (2018). Systematic review of synthetic computed tomography generation methodologies for use in magnetic resonance imaging-only radiation therapy. Int J Radiat Oncol Biol Phys.

[5] Wang G, Zhang Y, Ye X, Mou X, Verhaegen F (2019). Image quality assessment. Machine learning for tomographic imaging.

[6] Chow LS, Paramesran R (2016). Review of medical image quality assessment. Biomed Signal Process Control.

[7] Dowling J (2022). Image synthesis for MRI-only radiotherapy treatment planning.

[8] Boulanger M (2021). Deep learning methods to generate synthetic CT from MRI in radiotherapy: a literature review. Phys Medica.

[9] Li Y, Xu S, Lu Y, Qi Z (2023). CT synthesis from MRI with an improved multi-scale learning network. Front Phys.

[10] Largent A (2019). Pseudo-CT generation for MRI-Only Radiation Therapy Treatment Planning: comparison among Patch-Based, Atlas-Based, and Bulk Density Methods. Int J Radiat Oncol Biol Phys.

[11] Chourak H et al (2021) IEEE 18th international symposium on biomedical imaging (ISBI), 395–399, 2021. 10.1109/ISBI48211.2021.9433800

[12] Kemppainen R (2019). Assessment of dosimetric and positioning accuracy of a magnetic resonance imaging-only solution for external beam radiotherapy of pelvic anatomy. Phys Imaging Radiat Oncol.

[13] Chourak H (2022). Quality assurance for MRI-only radiation therapy: A voxel-wise population-based methodology for image and dose assessment of synthetic CT generation methods. Front Oncol.

[14] Hyuk Choi J (2023). Investigation of a water equivalent depth method for dosimetric accuracy evaluation of synthetic CT. Physica Med.

[15] Morris MD (1991). Factorial sampling plans for preliminary computational experiments. Technometrics.

[16] Cacuci DG, Ionescu-Bujor M, Navon IM (2003). Sensitivity and uncertainty analysis.

[17] Qian G, Mahdi A (2020). Sensitivity analysis methods in the biomedical sciences. Math Biosci.

[18] Adjeiwaah M, Garpebring A, Nyholm T (2020). Sensitivity analysis of different quality assurance methods for magnetic resonance imaging in radiotherapy. Phys Imaging Radiat Oncol.

[19] Barnard RC, Frank M, Krycki K (2017). Sensitivity analysis for dose deposition in radiotherapy via a Fokker-Planck model. Math Med Biology.

[20] Sosa-Marrero C (2021). Towards a reduced in silico model predicting biochemical recurrence after radiotherapy in prostate cancer. IEEE Trans Biomed Eng.

[21] Iooss B et al (2022) Sensitivity: global sensitivity analysis of model outputs. R package version 1.28.0. https://CRAN.R-project.org/package=sensitivity

[22] Campolongo F, Saltelli A, Cariboni J (2011). From screening to quantitative sensitivity analysis. A unified approach. Comput Phys Commun.

[23] Ojeda D (2014). Sensitivity analysis and parameter estimation of a coronary circulation model for triple-vessel disease. IEEE Trans Biomed Eng.

[24] Zhao B (2023). CT synthesis from MR in the pelvic area using residual transformer conditional GAN. Comput Med Imaging Graph.

[25] Tahri S (2022). A high-performance method of deep learning for prostate MR-only radiotherapy planning using an optimized Pix2Pix architecture. Phys Medica.

[26] Largent A (2019). Comparison of Deep Learning-Based and Patch-Based methods for Pseudo-CT generation in MRI-Based prostate dose planning. Int J Radiat Oncol Biol Phys.

[27] Liu Y (2019). Evaluation of a deep learning-based pelvic synthetic CT generation technique for MRI-based prostate proton treatment planning. Phys Med Biol.

[28] Wieser HP (2017). Development of the open-source dose calculation and optimization toolkit matRad. Med Phys.

[29] MacFarlane M, Hoover DA, Wong E, Battista JJ, Chen JZ (2020). Technical note: a fast inverse direct aperture optimization algorithm for volumetric-modulated arc therapy. Med Phys.

[30] Kamal R (2020). Efficiency of a novel non-monotonic segmented leaf sequence delivery of varian MLC for non-split IMRT fields. Rep Practical Oncol Radiother.

[31] Her EJ (2020). Voxel-level biological optimisation of prostate IMRT using patient-specific tumour location and clonogen density derived from mpMRI. Radiat Oncol.

[32] Nuti R, Martini G, Gennari C (1995). Age-related changes of whole skeleton and body composition in healthy men. Calcif Tissue Int.

[33] Thomas SJ (1999). Relative electron density calibration of CT scanners for radiotherapy treatment planning. Br J Radiol.

[34] Zurl B, Tiefling R, Winkler P, Kindl P, Kapp KS (2014). Hounsfield units variations: impact on CT-density based conversion tables and their effects on dose distribution. Strahlenther Onkol.

[35] Davis AT, Palmer AL, Nisbet A (2017). Can CT scan protocols used for radiotherapy treatment planning be adjusted to optimize image quality and patient dose? A systematic review. Br J Radiol.

[36] Podder TK, Fredman ET, Schatten H (2018). Advances in radiotherapy for prostate cancer treatment. Molecular & diagnostic imaging in prostate cancer: clinical applications and treatment strategies.

[37] Dowdell SJ, Metcalfe PE, Morales JE, Jackson M, Rosenfeld AB (2008). A comparison of proton therapy and IMRT treatment plans for prostate radiotherapy. Australas Phys Eng Sci Med.

[38] Chen Z, Yang Z, Wang J, Hu W (2016). Dosimetric impact of different bladder and rectum filling during prostate cancer radiotherapy. Radiat Oncol.

[39] Xiong Y (2022). Assessment of intrafractional prostate motion and its dosimetric impact in MRI-guided online adaptive radiotherapy with gating. Strahlenther Onkol.

[40] Spadea MF, Maspero M, Zaffino P, Seco J (2021). Deep learning based synthetic-CT generation in radiotherapy and PET: a review. Med Phys.

[41] Abdar M et al (2020) A review of uncertainty quantification in deep learning: techniques, applications and challenges, Available: http://arxiv.org/abs/2011.06225

[42] Hemsley M (2020). Deep generative model for synthetic-CT generation with uncertainty predictions.

[43] van den Berg CAT, Meliadò EF (2022). Uncertainty assessment for deep learning radiotherapy applications. Sem Radiat Oncol.

